# Decoding the Microcin J25 Biosynthetic Cluster: Modulation of the *mcjA* Promoter by the Novel Overlapping Gene *mcjX*

**DOI:** 10.3390/ijms27135741

**Published:** 2026-06-25

**Authors:** Emilse Masias, Juan I. Ramirez, Lucía Lanza, Jorge A. Lachenicht, María E. Vázquez, Leonardo Acuña, Carlos J. Minahk, Raul A. Salomón

**Affiliations:** 1 Instituto Superior de Investigaciones Biológicas (INSIBIO), CONICET-UNT, and Instituto de Química Biológica “Dr. Bernabé Bloj”, Facultad de Bioquímica, Química y Farmacia, UNT. Chacabuco 461, San Miguel de Tucumán T4000ILI, Argentina; emilse.masias@fbqf.unt.edu.ar (E.M.); juanigna.ramirez@gmail.com (J.I.R.); lulanza_522@hotmail.com.ar (L.L.); andres_lachenicht@hotmail.com (J.A.L.); 2 Unidad de Biotecnología y Protozoarios, Instituto de Patología Experimental “Dr. Miguel Ángel Basombrío”, Consejo Nacional de Investigaciones Científicas y Técnicas (CONICET), Universidad Nacional de Salta, Salta A4408FVY, Argentina; elisa.vazquez@conicet.gov.ar (M.E.V.); leonardo.a@conicet.gov.ar (L.A.)

**Keywords:** *Escherichia coli*, gene expression, MccJ25, green fluorescent protein

## Abstract

A comprehensive analysis of the microcin J25 (MccJ25) biosynthetic gene cluster revealed a previously uncharacterized 96-base pair overlapping gene, designated *mcjX*. This gene features a +1 reading frame shift relative to the primary sequence and encodes a 31-amino acid peptide. Notably, 53 nucleotides overlap with the 3′ terminus of the structural gene *mcjA*. Such significant overlaps are rare features in the *Escherichia coli* genome, highlighting the hidden complexity of microbial operon architectures. In this study, we demonstrate that *mcjX* is actively translated. Functional assays, including green fluorescent protein reporter systems, suggest that McjX acts as a negative regulator of the *mcjA* promoter, modulating MccJ25 expression. This discovery represents the first report of a regulatory mechanism mediated by an overlapping gene within a lasso peptide operon, providing new perspectives on how microbial genomes fine-tune the production of antimicrobial peptides through compact genetic organization.

## 1. Introduction

Microcin J25 (MccJ25) is a 21-amino acid antibacterial peptide of ribosomal synthesis (2107 Da), isolated in our laboratory in the early 90s from an intestinal strain of *Escherichia coli* [1,2]. The MccJ25 system consists of four genes: *mcjA*, *mcjB*, *mcjC* and *mcjD* [3]. The transcription of the structural gene *mcjA* and the *mcjBCD* operon is divergent [3]. The structural gene *mcjA* encodes a precursor of 58 amino acids, which is processed by the proteins McjB and McjC [4]. McjD is an ABC exporter ensuring both the secretion of the peptide and the self-immunity of the producer to MccJ25 [3,5]. MccJ25 is an antimicrobial peptide that has garnered significant interest for two key reasons: its unique structure and its unusual mechanism of action. This antibiotic inhibits bacterial RNA polymerase, but instead of targeting the active site, MccJ25 blocks the enzyme’s secondary channel, preventing the entry of nucleoside triphosphates. This original approach effectively shuts down the transcription process [6]. The structure is also particular: its three-dimensional structure resembles a bow or slipknot [7,8,9]. This compact folding gives the peptide a remarkable resistance to proteases, extreme temperature and pH, as well as denaturing agents [1]. Of note, even though it is resistant to most known proteases, it is sensitive to digestion by thermolysin and by elastase, a protease secreted by the pancreas [10,11].

The synthesis of MccJ25 is strongly dependent on the growth phase, being induced in the stationary phase by a lack of nutrients, in particular carbon and phosphate sources. These features have been studied by fusions of the *mcjA* gene with *lacZ*, measuring the activity of β-galactosidase under different conditions [12]. It has been demonstrated that the expression of this peptide depends on the concerted action of the nucleotide ppGpp, the integration host factor (IHF), and the leucine-responsive regulatory protein (Lrp) [12]. Despite that, the structure of the *mcjA* promoter (*PmcjA*) could not be determined in a definitive way so far. In fact, two possible -35/-10 pairs have been proposed: the first one dated from 1999, after the first genetic sequence analysis [13]. The -35 sequence proposed (TTGAtA) was close to the consensus, whereas the -10 was proposed considering a 17 bp spacer from that -35 box. Such a -10 sequence (gATtAT) was also highly likely since it has four out of six bases of the consensus sequence. Later, a second pair -35/-10 was proposed [12]. In this case, the -10 sequence (TAaAAT) was closer to the consensus, whereas the -35 sequence (aTctCA) was proposed to be located 17 bp upstream; it moves away from the consensus, though. To date, no detailed analysis of *PmcjA* has been conducted; consequently, both its definitive structure and the regulatory mechanisms preventing MccJ25 overexpression remain poorly understood. Fine-tuning the in vivo expression of antimicrobial peptides is critical, as elevated dosages of MccJ25 have been shown to increase intestinal permeability and trigger microbial dysbiosis in mice [14].

Since *PmcjA* seems to direct the expression of *mcjA* mainly in stationary phase, engineered MccJ25 gene clusters were designed for improving the peptide expression, such as the construction reported by Yu et al., with a codon optimization of *mcjA* under the PDZ93 promoter [15,16]. In addition, *mcjA* was expressed under the IPTG-inducible T5 promoter in James Link’s lab [17,18]. Further investigation by this research group involved the construction of the expression plasmid pJP62. In this vector, the *mcjA* and *mcjBCD* operons were cloned in a co-linear arrangement, driven by their respective native promoters. Surprisingly, *E. coli* strains carrying this construct exhibited a moderate increase in microcin expression [19]. The fact that an inversion of the operon resulted in improved expression was worthy of attention and led us to conduct a more detailed examination of the operon sequence. This re-analysis of the *mcjA* sequence uncovered a previously unannotated gene. This cryptic gene overlaps the 3′ terminus of *mcjA* and extends downstream in a +1 frameshift. Given this unique architecture and the fact that *PmcjA* remained uncharacterized, we sought to investigate its functional properties and elucidate the regulatory role this overlapping gene plays.

## 2. Results

### 2.1. The Overlapping Gene in the mcjA Locus

**Figure 1:**
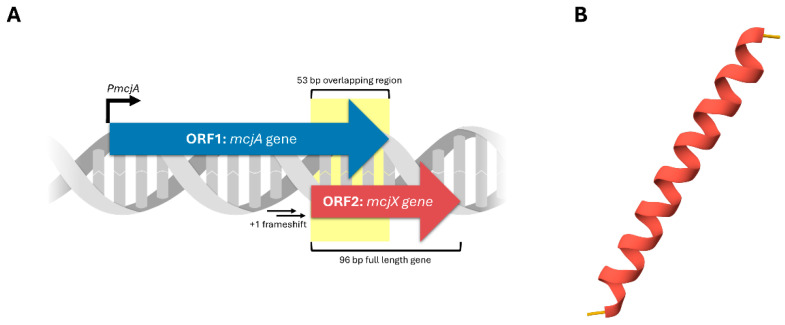
New identified ORF and its predicted product structure. (**A**) The figure shows the open reading frames downstream of *PmcjA*, revealing a significant overlap. The lengths of both the cryptic gene and the overlapping region are also indicated. (**B**) Predicted structure of McjX.

Using the NCBI ORF Finder, we analyzed the sequence downstream of *PmcjA*. This search identified two open reading frames (ORFs): ORF1, which corresponds to the *mcjA* gene, and a second, previously uncharacterized ORF, which was named *mcjX* (Figure 1A and Figure S1).

The *mcjX* ORF is a short, 96 bp sequence (including its stop codon) that encodes a 31-amino acid peptide. This sequence shows no homology to any known genes, but a putative Shine–Dalgarno motif suggests it is actively translated. We found that the *mcjX* ORF is in a different reading frame than *mcjA* and partially overlaps it, with 53 nucleotides extending into the 3′ end of *mcjA* and 43 nucleotides beyond its stop codon. If translated, the resulting amino acid sequence would be M^1^-C-L-S-I^5^-L-W-G-L-V^10^-H-L-Y-L-S^15^-M-A-D-I-L^20^-K-E-E-L-C^25^-H-F-V-Y-Q^30^-Q, with a molecular weight of 3697.44 Da and a theoretical isoelectric point of 5.27, computed using the Expasy server [20]. Structural predictions using AlphaFold suggest that McjX adopts an α-helical conformation (Figure 1B) [21].

### 2.2. mcjX Gene Is Translated

**Figure 2:**
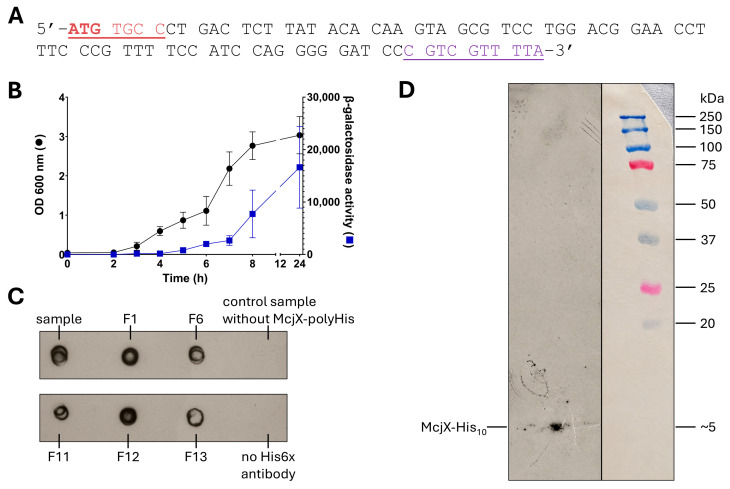
*mcjX* expression. (**A**) Sequence of the *mcjX*-*lacZ* fusion. The first two codons and one nucleotide of the third codon of *mcjX* are denoted in red, then the 5′ sequence of the TnlacZ is shown in black, and finally the sequence of *lacZ* is denoted in violet; (**B**) *mcjX*-*lacZ* expression during *E. coli* growth estimated by Miller units; (**C**) dot-blot analysis of McjX-polyHis fusion. The bacterial extract from pCHm3H10C-mcjX-bearing *E. coli* DH5α is denoted as “sample”. F1 to F6 correspond to the washing steps after loading the Ni-NTA column with the sample. F11–F13 correspond to the elution of McjX-polyHis with 300 mM imidazole; (**D**) Western-blot of McjX-polyHis is shown in the left panel, whereas molecular weight markers are shown in the right panel.

Since an uncharacterized open reading frame was recently identified, we sought to determine whether *mcjX* is actively expressed. As an initial approach, a translational *lacZ* fusion to *mcjX* was attempted. Most of the *lacZ* fusions obtained with the Tn5 insertion were in-frame with *mcjA* gene, but in one of them, the *lacZ* gene got in-frame with *mcjX*, evidenced by the DNA sequence analysis (Figure 2A). The resulting plasmid was called pMcx-LacZ and was used for analyzing the expression of McjX. Figure 2B shows the bacterial growth and the Miller units calculated from the β-galactosidase activity of the McjX-LacZ fusion. As can be seen, the kinetics of the expression of *mcjX-lacZ* is similar to the previously reported *mcjA-lacZ* expression [12]. This result strongly suggests that *mcjX* is indeed expressed and then translated into a peptide.

To validate this finding, *mcjX* was cloned and fused at its 3′ end with a poly-histidine-coding sequence. Dot-blot analysis showed that McjX was, in fact, translated. No signal was obtained in the absence of either the bacterial extract containing McjX-polyHis or the polyHis antibody (i.e., the dots observed were highly specific) (Figure 2C).

Furthermore, a noticeable band at the expected molecular weight was visible via Western blot (Figure 2D), confirming that McjX is indeed translated using its own Shine–Dalgarno sequence.

### 2.3. McjX Modulates MccJ25 Production

**Figure 3:**
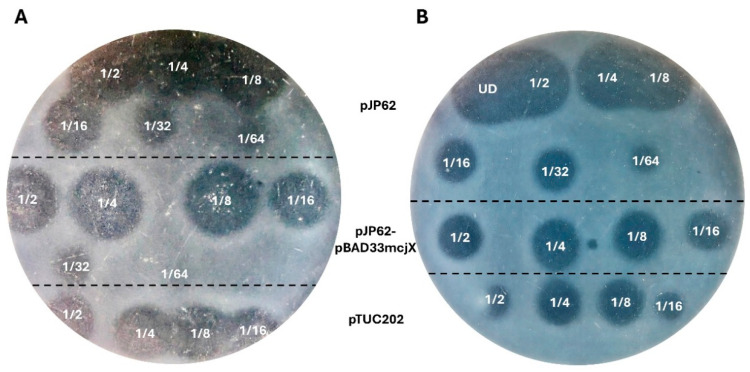
Expression of MccJ25 from pJP62 plasmid. (**A**) MccJ25 was titrated in the supernatants of *E. coli* DH5α (pJP62) and *E. coli* DH5α (pJP62, pBAD33mcjX) grown in the presence of glucose, (**B**) and in the presence of arabinose. MccJ25 production from *E. coli* DH5α (pTUC202) was included as a control (bottom panel). *E. coli* AB1133 was used as a sensitive strain. UD indicates the undiluted supernatant. This result is representative of three independent experiments.

As mentioned above, the plasmid pJP62 [19] has the *mcjA* and *mcjBCD* genes cloned in the same direction under their native promoters. Sequence analysis revealed that the *PmcjA*-*mcjA* inversion during the construction of pJP62 led to the truncation of the *mcjX* gene. This genetic alteration creates an ideal system for investigating the role of *mcjX* in the expression of MccJ25. For this purpose, *E. coli* DH5α bearing pJP62 plasmid was co-transformed with pBAD33-mcjX and cells were grown in minimal medium supplemented with either glucose or arabinose. Figure 3 clearly shows that microcin production by pJP62 was significantly lower upon addition of arabinose when *mcjX* was expressed in trans, compared to the production of the strain that only carried pJP62. MccJ25 production was almost identical in *E. coli* DH5α (pJP62) and *E. coli* DH5α (pJP62, pBAD33-mcjX) in the presence of glucose, which was expected since *mcjX* expression was repressed in this condition. In the last row, dilutions of the supernatant of a *E. coli* DH5α (pTUC202) culture carrying the integral MccJ25 system were spotted on both plates as a control.

**Figure 4:**
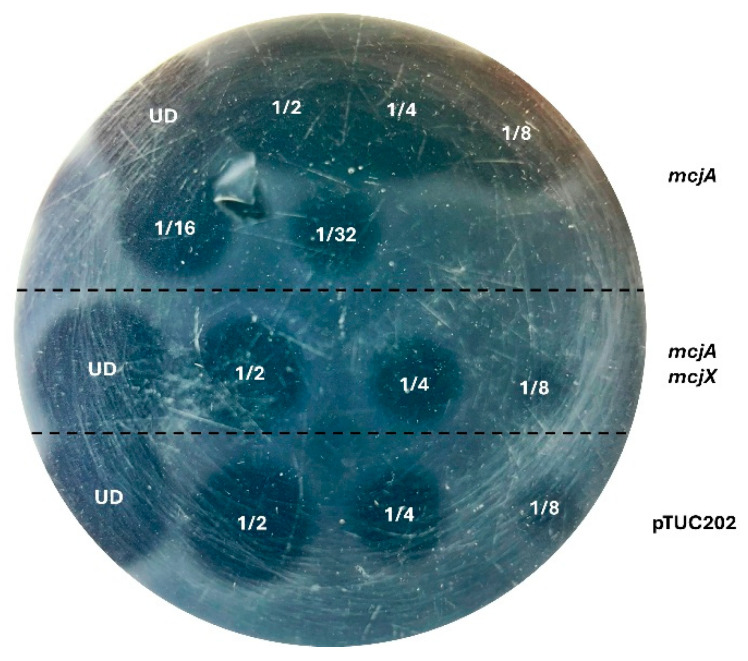
Inhibition of MccJ25 production by *mcjX* expression. Titration of MccJ25 activity in the presence or absence of *mcjX* is shown. *E. coli* AB1133 was used as a sensitive strain. UD indicates the undiluted supernatant. This result is representative of three independent experiments.

To confirm this finding, pBAD33-mcjA and pBAD33-mcjA-mcjX were co-transformed with pTUC341-7, a pBR322 derivative that allows cells to express *mcjB*, *mcjC* and *mcjD* but cannot produce mature MccJ25 since *mcjA* and *mcjX* are inactivated by a Tn5 insertion. In agreement with our previous results, the expression of *mcjX* alongside *mcjA* upon addition of arabinose led to a reduced activity compared to the activity verified when *mcjA* expression alone was induced with arabinose, as if the concomitant induction of *mcjX* would partly “turn off” microcin production. In fact, MccJ25 secretion was higher in the absence of the *mcjX* gene [DH5α (pTUC341-7, pBADmcjA)] (top of Figure 4) as compared to the secretion of MccJ25 in the strain expressing *mcjA* and *mcjX* (center of Figure 4). A supernatant of DH5α (pTUC202) was titrated as a control (bottom of Figure 4). In this last plasmid, the entire MccJ25 system is present and, therefore, *mcjX* is active. As expected, no expression of MccJ25 was observed in the presence of 0.6% glucose, a condition where the *araBAD* promoter is repressed [22].

On top of that, the complete MccJ25 system was amplified from the 3′ end of *mcjD* to 13 bp beyond the stop codon of *mcjA*, excluding the final nine codons of *mcjX,* as described in Materials and Methods. The antimicrobial activity due to the production and secretion of MccJ25 is shown in Table 1. The activity of MccJ25 was higher in those bacteria bearing plasmids encoding the truncated *mcjX* gene, particularly in M63 minimal medium. Noteworthy, the cloning of the MccJ25 system in a high-copy number plasmid such as pUC18 did not have a proportional impact on the production of MccJ25. In fact, beyond the effect of McjX, one can also compare the production of MccJ25 when cells have a low copy number plasmid such as pTUC202 (10–12 copies per cell), the pTUC341, a plasmid derived from pBR322 with ~20 copies per cell, and pTUC346, derived from pUC18 with more than 700 copies per cell. No significant correlation was observed even when comparing plasmids lacking *mcjX*. For instance, while cells transformed with the pBR322-derived plasmid exhibited an MccJ25 activity of 32,000 AU/mL, this value only increased twofold when using the pUC18-derived vector (pTUC346woX) in the same condition.

These results prompted a more detailed investigation of *PmcjA* and its regulatory mechanisms.

### 2.4. The mcjA-mcjX Operon Is Controlled by a Promoter with an Extended -10 Sequence

**Figure 5:**
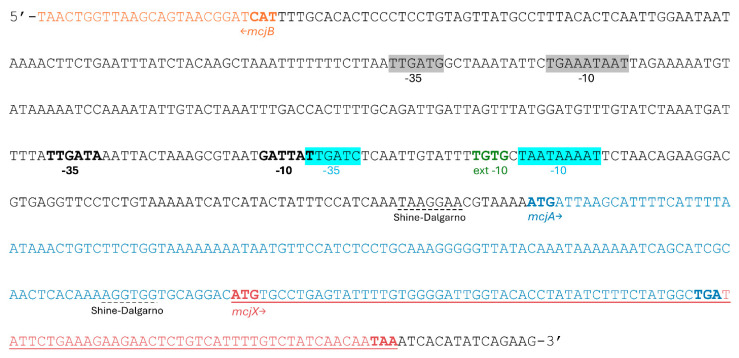
Sequence analysis of the *mcjA-mcjB* intergenic region. The complete intergenic region is shown, with the *mcjA* sequence indicated in blue and its respective start and stop codons in bold blue. The extended -10 promoter motif is highlighted in bold green. The *mcjX* gene is underlined, with its start and stop codons shown in bold red. The initial 26 nucleotides of the complementary *mcjB* sequence appear in orange. The first suggested -35/-10 elements of *PmcjA*, as originally proposed [13], are denoted in bold black.

BPROM was run and the first hit was found, with a linear discriminant function (LDF) score of 7.5. This first proposed promoter has a -10 element (TGAAATAAT) at position 110 of the intergenic region and a -35 element (TTGATG) at position 96 (both boxes are highlighted in gray, Figure 5). A second possibility was a promoter with a LDF score of 4.82, which is closer to that predicted previously by Chiuchiolo et al. (2001) [12]. The -10 element (TAATAAAAT) was found at position 266 of the intergenic region and the -35 element (TTGATC) at position 240 of the intergenic region. This predicted promoter is highlighted in cyan in Figure 5. Of note, if the last prediction were correct, the promoter would have an extended -10 motif, which is shown in green fonts (“TG” located at positions -14/-15). Moreover, there is another “TG” at positions -16/-17 that could also have an impact on the activity of *PmcjA* [23]. As mentioned above, a second ORF could be found. This 96 bp gene, called *mcjX*, has a +1 reading frame shift related to *mcjA* and its own Shine–Dalgarno sequence associated.

### 2.5. PmcjA Region Can Be Shortened Five Times Without Losing Its Activity

**Figure 6:**
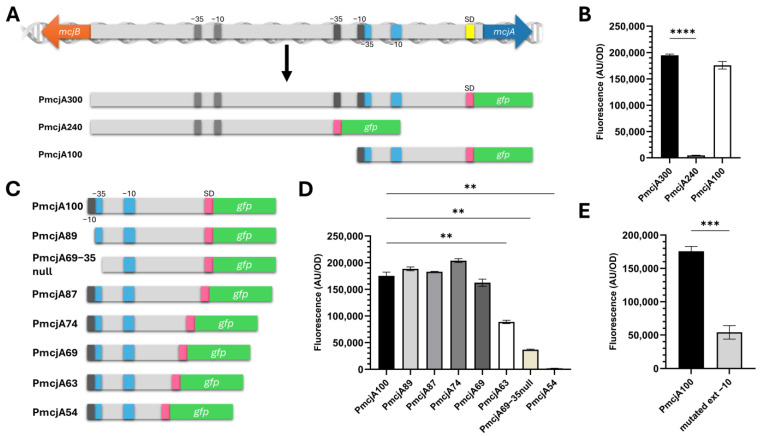
Analysis of *PmcjA*. (**A**) The intergenic region and the two pairs of predicted -35/-10 elements by BPROM are depicted in gray and cyan; (**B**) the promoter activity of these fragments is shown; (**C**) deletion analysis of PmcjA100 is summarized; (**D**) promoter activity of each construct; (**E**) change in PmcjA100 activity upon mutation of the extended -10 sequence. The Shine–Dalgarno sequence from pProbe-NT is shown in pink, whereas a Shine–Dalgarno sequence from the MccJ25 system is shown in yellow. One-way ANOVA and Dunnett’s multiple comparisons test ((**B**) and (**D**) compared to control groups denoted in black bars) were performed using GraphPad Prism, which assigned a score from ** to **** to significantly different groups (*p* < 0.05). Unpaired *t*-test was performed using GraphPad Prism for (**E**).

As a first approach, the complete intergenic region (PmcjA300) was cloned into the promoterless plasmid pProbe-NT, as well as a 240 bp fragment (PmcjA240) that starts from the beginning of the intergenic region, and another fragment that includes the last 114 bp of the intergenic region located just upstream the Shine–Dalgarno sequence of *mcjA* (PmcjA100), as shown in Figure 6A. The latter fragment retains the same activity as the complete intergenic region. Conversely, there was no production of green fluorescent protein (GFP) when the 240 pb fragment was cloned as a possible promoter in the multiple cloning site of pProbe-NT (Figure 6B). This result confirms that *PmcjA* is located closer to the structural gene, ruling out the first prediction of BPROM. Furthermore, since PmcjA100 includes the -10 box but excludes the -35 box of the promoter predicted by Solbiati et al. (1999) [13], the involvement of this putative promoter in *mcjA* expression also becomes highly unlikely.

Afterward, a series of *PmcjA* variants was constructed (Figure 6C). The sequence of each promoter variant is shown in Table S2. The fluorescence of GFP expressed under the control of these promoters was recorded in bacteria grown for 24 h at 37 °C. As mentioned above, PmcjA100 includes the -10 sequence of the first proposed promoter. This element proved to be non-essential since PmcjA89, which lacks this -10 box, displayed the same potency as PmcjA300 and PmcjA100 (Figure 6D). On the other hand, the GFP production was greatly reduced when the -35 element proposed was deleted (PmcjA69-35null, Figure 6D), strongly suggesting that the second pair of -35/-10 elements proposed by BPROM for *PmcjA* is more likely involved. PmcjA69 represents the corresponding intact promoter containing both the proposed -35 and -10 elements and serves as the reference control for promoter activity for PmcjA69-35null. Once we ruled out the first -35/-10 elements, we focused on investigating the activity of shorter variants of the promoter. As can be observed in Figure 6D, the expression of the GFP remained unchanged up to a 69 bp fragment. As a matter of fact, there was a significant reduction with the 63 bp fragment, and the expression was completely abrogated with the 54 bp fragment. Interestingly, the latter construct includes 10 bases beyond the -10 element (i.e., it may contain the transcription start point) and yet no expression was found. Since the initially proposed -10 element does not influence *gfp* gene expression, most promoters were designed with 12 bases upstream of the -35 element because it enhanced the efficiency of fragment purification.

On top of that, we were able to mutagenize the extended -10 sequence. Indeed, there was a significant reduction in the fluorescence upon changing the “TG” present upstream of the -10 box (Figure 6E). This result confirms the position of the -10 sequence and the presence of an “extended -10” in *PmcjA*.

### 2.6. McjX Reduces the Activity of PmcjA Variants

**Figure 7:**
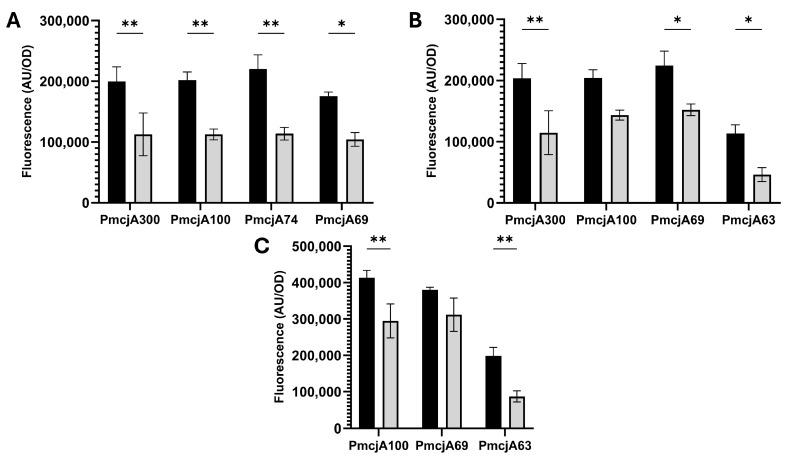
*PmcjA* activity in response to *mcjX* expression. (**A**) *mcjX* was expressed in trans from a pACYC-duet vector under the control of an IPTG-inducible T7 promoter; (**B**) *mcjX* was expressed in trans from a pUC18-derived plasmid under the control of a *PmcjA* derivative; (**C**) *mcjX* was expressed in cis under the control of three *PmcjA* variants using a GFP reporter plasmid. Black columns represent control conditions (absence of *mcjX* expression), while gray columns indicate GFP fluorescence levels during *mcjX* expression. Two-way ANOVA and Šídák’s multiple comparisons test (compared to control groups denoted in black bars) were performed using GraphPad Prism, which assigned a score from * to ** to significantly different groups (*p* < 0.05).

*mcjX* was cloned into pACYCDuet-1 under the control of the T7 promoter, and *E. coli* BL21 was co-transformed with this plasmid and plasmids derived from pProbe-NT bearing *PmcjA* variants. Bacteria grew for 24 h at 37 °C in M63 medium and *mcjX* was induced from the beginning of the incubation with 50 μM IPTG. There was a 35–50% reduction in the GFP fluorescence for all the promoters tested (Figure 7A).

In parallel experiments, *mcjX* was also cloned into pUC18 under the control of PmcjA69, and *E. coli* was co-transformed with pProbe plasmids bearing variants of *PmcjA*. As observed with the T7 promoter, the expression of *mcjX* under the microcin promoter also reduced the expression of *gfp* (Figure 7B). Since a *PmcjA* variant was used instead of the T7 promoter, this approach did not need the addition of any inducer to the culture media. The following step assessed the effect of *mcjX* cloned into the same vector as *gfp*, under the control of the same *PmcjA* derivative. The role of *mcjX* as a negative regulator was observed once again (Figure 7C). The induction of *mcjX* by IPTG did not confer resistance to exogenous MccJ25 (Figure S4).

## 3. Discussion

A common finding in bacterial genomes is that about one-third of their genes overlap, though typically by only a few base pairs. Most overlapping genes are unidirectional. This close arrangement is a key feature of how bacteria efficiently use their limited DNA [24]. Long, non-trivial overlaps, such as the one presented in this work (a 53-nucleotide overlap), are rare, and in *E. coli*, long overlaps have only been confirmed in a few pairs of genes [25]. Most studies of overlap in bacterial genomes indicate that it has a regulatory function, allowing overlapping genes to be transcriptionally and translationally coregulated, as occurs with the transcriptional regulation of *mcjA* and *mcjX*. Even though most studied gene overlaps are chromosomal, *mcjA* and *mcjX* are plasmid genes. Our results suggest that the partial inhibition of *PmcjA* is most likely due to the *mcjX* product. Although the regulatory mechanism is still hypothetical, several observations support this idea. When the *mcjX* gene was expressed in trans from an inducible T7 promoter, the activity of the *PmcjA*-*gfp* fusion, which reflects the transcription from the structural gene, was inhibited. The same effect was observed when *mcjX* was expressed in cis. On the other hand, a deletion of nine codons from the 3′ end of *mcjX* significantly increased the production of MccJ25. It is very likely that the truncated McjX is inactive or its function is seriously affected, at the very least. In agreement with our findings, the deletion of the 3′ end of *mcjX* due to an inversion of the *mcjA* sequence during the construction of the pJP62 plasmid in Dr. Link’s lab stimulated the production of the microcin [19]. If McjX directly inhibited *PmcjA*, this would represent a form of autogenous regulation, a common mechanism in which a gene product controls its own expression and that of other genes within its operon [26]. While some autogenous control mechanisms operate at the post-transcriptional level by binding mRNA, our findings with the *PmcjA*-*gfp* fusion suggest that McjX might mediate repression at the transcriptional level. However, direct interaction between McjX and the *PmcjA* region would be highly unlikely because of the low isoelectric point of this helical peptide. For the same reason, an interaction between McjX and ppGpp, which has been linked to MccJ25 production, is also improbable. Alternatively, McjX might interact with IHF and Lrp, both of which have been proposed as potential regulators of the *PmcjA* promoter [12]. However, because the identified binding sites for these transcription factors are located upstream of PmcjA100 and the other promoter variants, this possibility remains equally unlikely.

Regarding the biological role of *mcjX*, it is plausible to hypothesize that the downregulation of *mcjA* serves as a protective mechanism to prevent the toxic intracellular MccJ25 accumulation. However, experimental evidence suggests otherwise: when *mcjX* is disrupted, as seen in plasmids pTUC202woX, pTUC341woX and pTUC346woX, the resulting marked increase in both intracellular production and extracellular secretion does not yield observable cellular toxicity (Table 1 and Figure S5). Furthermore, the significant increase in MccJ25 production following the truncation or deletion of *mcjX* indicates that the exporter McjD and the biosynthetic enzymes, McjB and McjC, are not operating at physiological saturation. Consequently, *mcjX* appears to be non-essential for the viability of MccJ25-producing cells, at least under standard laboratory conditions. If the primary objective is to maximize MccJ25 yield, the strategic deletion of *mcjX* is advisable. In addition, any mutation or truncation of *mcjX* could impact the mRNA stability or its secondary structure. The proposed deletion of *mcjX* aligns with efforts by Dr. Link to enhance production by modifying or inverting the native promoter. Similarly, Zhang et al. demonstrated the importance of expression analysis and promoter optimization when producing MccJ25 and MccY in *Bacillus subtilis* [27].

In summary, analysis of the nucleotide sequence downstream of *PmcjA* identified an overlapping gene, *mcjX*. This gene, which is translated via a +1 frameshift, encodes the McjX peptide. When *mcjX* was induced, a significant decrease in GFP fluorescence and MccJ25 production was observed, strongly suggesting that its product functions as a negative regulator of *PmcjA*. On top of that, the deletion of the last nine codons of *mcjX*, which results in the absence of the intact McjX peptide, was also associated with increased MccJ25 production (Table 1). This observation suggests that the peptide itself, rather than solely structural alterations in the transcript, contributes to the observed regulatory effect. Nevertheless, we cannot currently exclude the possibility that the overlapping RNA sequence also plays a role in this process. Therefore, further studies will be required to elucidate the precise molecular mechanism underlying McjX-mediated regulation. Characterizing *PmcjA* and its regulatory mechanisms is crucial for its future use in heterologous protein expression. By understanding how this promoter is controlled, we can harness its power to precisely regulate the production of other proteins, a key process in future biotechnological applications.

*mcjX* represents the first characterized overlapping gene involved in the regulation of a lasso peptide operon. Preliminary analysis allowed us to identify similar putative open reading frames within the klebsidin (*Klebsiella pneumoniae*) and acinetodin (*Acinetobacter gyllenbergii*) biosynthetic clusters, which suggests that this regulatory mechanism may be a conserved feature in class II lasso peptides (Figure S6). These genetic systems were first identified and described by Metelev et al., starting from a detailed bioinformatic analysis using McjB as a bait [28]. A putative ORF encoding a 31-amino acid peptide was identified in the acinetodin cluster. This ORF, designated *aciX*, is located close to *aciA* and it has a 55-base pair overlap with the 5′ terminus of *aciB*. Moreover, the klebsidin cluster contains an overlapping gene that encodes a 21-amino acid peptide, termed *kleX*, which exhibits a 53-base pair overlap with its corresponding lasso peptide structural gene sequence, *kleA*. Notably, despite the lack of primary sequence homology with McjX, both AciX and KleX are predicted by AlphaFold to adopt α-helical structures [21]. The structural similarity between these peptides, along with the location of the ORFs that encode them, suggests a potentially conserved functional role in lasso peptide regulation, a hypothesis that warrants further experimental validation.

## 4. Materials and Methods

### 4.1. Bacterial Strains and Growth Conditions

*E. coli* DH5α (fhuA2 lac(del)U169 phoA glnV44 Φ80′ lacZ(del)M15 gyrA96 recA1 relA1 endA1 thi-1 hsdR17), *E. coli* AB1133 (thr-1 leuB6 Δ(gpt-proA)62 hisG4 argE3) and *E. coli* BL-21 (DE3) (ompT hsdSB(r_B_^−^ m_B_^−^) gal dcm λDE3) were routinely grown at 37 °C in Luria–Bertani (LB) broth and M63 minimal medium. M63 medium was supplemented with 0.2% glucose, 1 mM MgSO_4_, 0.2% casamino acids and 1 μg/mL vitamin B1. Solid media contained 1.6% agar. When needed, the media were supplemented with 5-bromo-4-chloro-3-indolyl-β-d-galactopyranoside (X-Gal) (40 μg/mL), 50 μg/mL kanamycin, 100 µg/mL ampicillin, 30 μg/mL chloramphenicol or 100 µg/mL isopropyl ß-D-1-thiogalactopyranoside (IPTG). Growth was monitored by measuring optical density (OD) at 600 nm and the fluorescence was measured in both an ISS PC1 spectrofluorometer (Champaign, IL, USA) and Perkin–Elmer LS-55 fluorometer (Shelton, CT, USA), setting the excitation wavelength at 470 nm and the emission wavelength at 510 nm.

### 4.2. Bioinformatic Analysis

The MccJ25 genetic system was analyzed with NCBI ORF Finder https://www.ncbi.nlm.nih.gov/orffinder/ (accessed on 1 February 2026) and the promoter was screened with BPROM to identify the σ70 promoter with a minimum LDF score threshold of 0.2 [29].

The preliminary analysis of the klebsidin and acinetodin operons was also performed using NCBI ORF Finder on the sequences of *Klebsiella pneumoniae* 4541-2 and *Acinetobacter gyllenbergii* CIP 110306, respectively https://www.ncbi.nlm.nih.gov/ (accessed on 16 April 2026).

### 4.3. mcjX Translation

To investigate whether *mcjX* was translated or not, two strategies were followed: a construction of a translational *lacZ* fusion to *mcjX* and the fusion of a poly-histidine coding sequence to the 3′ end of *mcjX*. The fusion of *lacZ* to *mcjX* was carried out as described previously [12]. Briefly, the pTUC202 plasmid was transformed into *E. coli* CC170, which carries the transposon TnlacZ. Cells were grown for up to 6 h. Then, TnlacZ transpositions were selected by plating bacteria on LB plates containing kanamycin and chloramphenicol. Plasmid DNA was extracted from a pool and used to transform *E. coli* CC118, a strain that has the ΔlacX74 deletion. The transformed cells were selected as blue colonies on LB plates containing kanamycin, chloramphenicol and X-Gal. Plasmid DNA was purified from those colonies that had lost MccJ25 production and the *mcjA*-*mcjX* region was sequenced by Sanger in order to see whether or not one of them had the *mcjX-lacZ* fusion (CERELA-CCT NOA Sur).

For the poly-histidine fusion construct, *PmcjA*-*mcjX* fragment was amplified from pProbe-PmcjA101-mcjX plasmid, avoiding the *mcjX* stop codon. This fragment was cloned into the shuttle plasmid pCHm3H10C, which has a sequence encoding a 10-histidine tail that is located just after the multiple cloning site [30]. Therefore, C-terminal poly-His fusions can be made with the protein of interest. Since the fragment was cloned between HindIII and XbaI sites, the yeast promoter present in pCHm3H10C was removed and replaced with the PmcjA101-*mcjX* construction. In a parallel cloning experiment, *mcjX* gene was ligated to the same plasmid but without excluding its stop codon to express *mcjX* without a C-terminal poly-His tag.

Plasmids were isolated with the Wizard^®^ Plus SV Minipreps DNA Purification System (Promega, Madison, WI, USA). Digestions with restriction endonucleases (New England Biolabs), ligation with T4 DNA ligase (New England Biolabs, Ipswich, MA, USA), heat-shock transformation of chemically competent bacteria, and agarose gel electrophoresis were carried out as described elsewhere [31].

### 4.4. β-Galactosidase Assays

For β-galactosidase activity measurements, cells were permeabilized with sodium dodecyl sulfate (SDS) and chloroform. The reactions started upon addition of o-nitrophenyl-β-D-galactoside (ONPG) as the substrate, and they were stopped with Na_2_CO_3_. OD was recorded at 420 nm and 550 nm. The activity was reported in Miller units [32]. The assays were repeated at least twice for each sample.

### 4.5. Western-Blot Analysis

*E. coli* DH5α transformed with pProbe-PmcjA101-mcjX plasmid were grown in 50 mL LB medium supplemented with ampicillin, with constant shaking at 37 °C till stationary phase. Then, cells were centrifuged at 8000× *g* and washed twice with saline solution. Then, cells were lysed by repeated freeze–thaw cycles, followed by sonication. Unbroken bacteria and cellular debris were discarded upon centrifugation at 15,000× *g* for 15 min. McjX-polyHis protein was purified by immobilized metal affinity chromatography using nickel-nitrilotriacetic acid (Ni-NTA) resin (Roche, Mannheim, Germany). Soluble proteins released from *E. coli* upon sonication were supplemented with imidazole to reach a final concentration of 10 mM. This solution was loaded in a Ni-NTA column pre-equilibrated with 10 mM imidazole. The column was washed with 5 volumes of 10 mM imidazole, pH 7 and then McjX-polyHis was eluted with 5 volumes of 300 mM imidazole, pH 7. The eluate was concentrated under vacuum with a Savant SpeedVac (Thermo Fisher Scientific, Waltham, MA, USA).

McjX-polyHis was electrophoresed in a 12% SDS-polyacrylamide gel, and then transferred to an Immobilon-P polyvinylidene difluoride (PVDF) membrane (Millipore, Burlington, MA, USA). The membrane was blocked for 1 h in Tris-buffered saline containing 5% nonfat milk, pH 7. After blocking, the membrane was washed three times with Tris-buffered saline supplemented with 0.05% Tween 20 (TBST). It was then incubated overnight with a 1:100 dilution of anti-polyHis at 4 °C and then incubated for 1 h with the secondary antibody (1:2000) in the blocking buffer at room temperature with gentle agitation. Immunoreactivity was detected by ECL Plus Western blotting detection system (GE Healthcare Life Sciences, Pittsburgh, MA, USA), exposing the PVDF membranes on X-ray film (GE Healthcare Life Sciences, Pittsburgh, MA, USA).

### 4.6. Expression of MccJ25 by the Natural System but in the Absence of the Newly Discovered Open Reading Frame

A fragment of 4.5 kb from the 3′ end of *mcjD* to 13 bp beyond the stop codon of *mcjA* was amplified with Pfu (Genbiotech, CABA, Buenos Aires, Argentina) to minimize errors. In this way, a truncation of the proposed second open reading frame took place, with the last nine codons of this gene out of the amplified fragment. Then, pBR322, pACYC184 and pUC18 plasmids, as well as the PCR fragment, were digested with HindIII and BamHI and ligated. The resulting plasmids were pTUC341woX, pTUC202woX and pTUC346woX. The corresponding controls were pTUC341, pTUC202 and pTUC346, plasmids that do not have any truncated sequence [13]. Cells were grown in either LB or M63 medium till stationary phase and cell-free supernatants were collected by centrifugation at 10,000× *g* for 10 min at 4 °C. Serial dilutions were performed to assess the MccJ25 production in a spot-on-lawn assay. The antimicrobial activity was expressed as arbitrary units per milliliter (AU/mL), defined as the reciprocal of the highest serial two-fold dilution showing a clear zone of growth inhibition of the indicator strain per milliliter of sample (supernatant).

### 4.7. PmcjA Analysis

The 340 bp *mcjA*-*mcjB* intergenic region, where *PmcjA* is, as well as shorter fragments of it were amplified by PCR with the GoTaq^®^ G2 DNA Polymerase (Promega, Madison, WI, USA), using pTUC202 as the template [4]. PCR products were purified by the Wizard^®^ SV Gel and PCR Clean-Up System (Promega). For the purification of fragments shorter than 100 bp, 50 μL PCR solution was mixed with 50 μL membrane binding solution from the kit and 300 μL of absolute ethanol prior to transferring the prepared PCR product to the SV minicolumn. The *PmcjA* fragments were cloned into pProbe-NT promoterless vector (Addgene #37818) between HindIII and BamHI sites. Primers used in this study are listed in Table S1.

The apparent extended -10 sequence was tested using the megaprimer method. To generate the megaprimer, the first amplification was programmed with 5 cycles, using the mutagenic primer at a final concentration of 20 nM and a flanking primer at a concentration twenty times lower. The final extension step of this first PCR was set at 35 min to ensure that all the partial products were converted to full-length products [33]. The second round of PCR was carried out upon addition of the other flanking primer and consisted of 25 cycles with a final extension of 10 min at 72 °C. Finally, the mutagenized promoter fragment was reamplified with the flanking primers and cloned into pProbe-NT plasmid between HindIII and BamHI sites.

### 4.8. mcjX Expression

For expression of the *mcjX* gene in trans, *mcjX* was amplified by PCR with the GoTaq^®^ G2 DNA Polymerase (Promega), using once again pTUC202 as the template [4]. Then, *mcjX* was cloned into pACYCDuet-1 plasmid between SalI and HindIII sites, resulting in the plasmid pACYCDuet-mcjX. *E. coli* BL21 cells were transformed with this plasmid and competent cells were prepared, which, in turn, were co-transformed with the pProbe-PmcjA derivatives. The co-transformed cells were resistant to both kanamycin and chloramphenicol and the induction of *mcjX* was achieved by adding 50 μM IPTG from the beginning since *mcjX* expression got under the control of the T7 promoter.

*mcjX*, *mcjA* and *mcjA*-*mcjX* were also cloned into the pBAD33 plasmid, a derivative of the pACYC184 vector. The genes were cloned between SalI and BamHI sites and their expression was controlled by the inducible *araBAD* promoter. Afterward, *E. coli* DH5α cells bearing pTUC341-7 plasmid were co-transformed with these constructs. Of note, pTUC341-7 is a pBR322 derivative, which contains a HindIII-SalI fragment that includes all the genes of the MccJ25 system but *mcjA* and *mcjX* are inactivated by a Tn5 insertion. Hence, cells transformed with this plasmid alone cannot express MccJ25, but pTUC341-7 can complement plasmids carrying *mcjA* for producing mature MccJ25. Alternatively, *mcjA* and *mcjA*-*mcjX* genes were cloned in pBR322 plasmid between HindIII and EcoRI sites under the PmcjA69 variant. *E. coli* DH5α bearing pTUC203-6 plasmid were co-transformed with these constructs. The latter plasmid is a pACYC184 derivative that bears the *mcjBCD* genes and complements plasmids carrying *mcjA* for the expression of mature MccJ25.

For expression of *mcjX* in cis, *mcjX* was ligated to pProbe-PmcjA101, pProbe-PmcjA69 and pProbe-PmcjA63 plasmids between KpnI and EcoRI sites (i.e., *mcjX* was cloned between the variants of *PmcjA* and the *gfp* gene). Notably, the *mcjX* fragment that was amplified by PCR included 30 bases located immediately upstream of this gene, ensuring the inclusion of its putative Shine–Dalgarno sequence.

### 4.9. Assay of Antibiotic Activity

For identifying MccJ25-producing colonies, each blue colony found in the Tn5 insertion assay was plated in duplicate on LB medium. Plates were incubated overnight at 37 °C to let bacteria express and secrete MccJ25. Then, one of the plates was exposed to chloroform vapors to kill all bacteria. Afterward, molten soft agar (0.6%) was inoculated with *E. coli* AB1133, a hypersensitive strain, poured onto the plate and incubated for 16 h at 37 °C. Those colonies that were unable to form a clear inhibition zone were selected and subsequently, the *mcjA* region was sequenced.

On the other hand, a spot-on-lawn assay was conducted to estimate the influence of the *mcjX* gene on the MccJ25 production. *E. coli* cells were grown in M63 minimal medium. Then, supernatants were collected by centrifugation at 10,000× *g* for 10 min. The dilutions of each supernatant were spotted onto the agar plate. The indicator strain *E. coli* AB1133 was either evenly spread onto agar plates using sterile cotton swabs or diluted in molten soft agar (0.6%) and poured onto the Petri dishes. The plates were incubated overnight at 37 °C and examined for growth inhibition zones. Antimicrobial activity assays were independently performed at least three times, yielding comparable results.

### 4.10. GenAI Use

The graphical abstract was generated using Gemini Pro.

### 4.11. Statistical Analysis

Experiments were performed in at least three independent biological replicates. Data are presented as mean ± standard deviation (SD). Statistical analyses and graphics were performed using GraphPad Prism software version 10.4.0 (San Diego, CA, USA). Differences were considered statistically significant at *p* < 0.05.

## 5. Conclusions

The present study reports the identification and function of *mcjX*, a novel cryptic gene overlapping the *mcjA* locus of the microcin J25 (MccJ25) system. We provide definitive evidence that *mcjX* is actively translated via a +1 frameshift into a 31-amino acid peptide that acts as a transcriptional repressor of *PmcjA*. Our findings suggest a unique autogenous regulatory mechanism where McjX limits MccJ25 production, at least partially; consequently, the disruption or truncation of *mcjX* significantly enhances microcin yields without inducing cellular toxicity. Furthermore, our precise mapping of the *PmcjA* minimal functional region and its extended -10 motif offers new insights into the Mccj25 system regulation. Although our results demonstrate that the canonical -14/-15 TG motif enhances *PmcjA* activity, the potential contribution of the -16/-17 TG motif remains an interesting aspect that warrants further investigation. Previous studies have suggested that this upstream motif may exert a synergistic effect when present together with the canonical -14/-15 TG element, thereby further strengthening promoter activity [23]. By leveraging the characterized *PmcjA* and lifting McjX-mediated repression, this system can be harnessed as a highly efficient, customizable platform for scaling up the production of MccJ25 and other valuable heterologous proteins. However, despite clear evidence of inhibition, the biological driver of this regulatory interaction and its molecular basis have yet to be determined.

## Figures and Tables

**Table 1:** MccJ25 production, with or without *mcjX,* using vectors with different copy numbers.

Plasmid	MccJ25 Activity in AU/mL	
	LB Medium	M63 Medium
pTUC202	450 ± 252	1400 ± 400
pTUC202woX	1800 ± 1007	14,400 ± 8053
pTUC341	450 ± 252	4000 ± 1600
pTUC341woX	1800 ± 1007	32,000 ± 12,800
pTUC346	1000 ± 400	4800 ± 1848
pTUC346woX	2000 ± 800	64,000 ± 25,600

Note: the activity values shown in the table are mean ± standard deviation.

## Data Availability

The data sets generated during and/or analyzed during the current study are available from the corresponding authors upon reasonable request.

## Supplementary Materials

The following supporting information can be downloaded at: https://www.mdpi.com/article/10.3390/ijms27135741/s1.

## Abbreviations

The following abbreviations are used in this manuscript:

AU/mL	arbitrary units per milliliter
GFP	green fluorescent protein
IPTG	isopropyl ß-D-1-thiogalactopyranoside
LDF	linear discriminant function
MccJ25	Microcin J25
Ni-NTA	nickel-nitrilotriacetic acid
ORF	open reading frame
