# Supplementary material for: Decoding the Microcin J25 Biosynthetic Cluster: Modulation of the mcjA Promoter by the Novel Overlapping Gene mcjX
Source: Int J Mol Sci. 2026 Jun 25;27(13):5741. doi: 10.3390/ijms27135741 (PMC13361873; doi:10.3390/ijms27135741)
Supplement: Supplementary file 1 [file ijms-27-05741-s001.zip › ijms-4315472-supplementary.pdf]

Table S1. Primers used in this study.

Primer	Sequence (5′ to 3′)
PromcjA300pbF	AGGAAGCTTATTTTGCACACTCCCTCCTG
PmcjAHindIIIF101	AGGAAGCTTTTGATCTCAATTGTATTTTGTG
PromcjA100pbF	AGGAAGCTTGCGTAATGATTATTGATCTC
PmcjABamHIR240	ACGGATCCATAATCATTACGCTTTAGTAATTTATC
pProbeFnewXhoI	CCGCTCGAGGTCACACTGCTTCCGGTAG
PmcjABamHIR89	ACGGATCCATTTGATGGAAATAGTATGATG
PmcjABamHIR87	ACGGATCCGTATGATGATTTTTACAGAG
PmcjABamHIR74	ACGGATCCTACAGAGGAACCTCACGTCC
PmcjABamHIR69	ACGGATCCAGGAACCTCACGTCCTTCTG
pProbeBamHIR63	ACGGATCCCTCACGTCCTTCTGTTAG
PmcJABamHIR54	ACGGATCCTTCTGTTAGAATTTTATTAG
PmcjA35nullXhoF	AGCTCGAGGTATTTTGTGCTAATAAAATTC
primermutext	TTGTATTTGACGCTAATAAAATTC
mcjAKpnIF	GTGGTACCAATCATCATACTATTTCCATC
mcjAEcoRIR	GGTGAATTCAGAATATCAGCCATAGAAAG
mcjXKpnIF	GTGGTACCCATCGCAACTCACAAAAAG
mcjXEcoRIR	GGTGAATTCCTGATATGTGATTTATTGTTG
mcjXXbaInoSTOP	CCTCTAGACCTTGTTGATAGACAAAATGACAG
A3Hindlll	GGTCCCAAGCTTCTGATATGTGATTTATTGTTG
P4Hindlll	GGTCCCAAGCTTTTCTTTCAGAATATCAGCCATAGAAAG
PmcjXSalI	GGTGGTGTCGACATCAGCATCGCAACTCACAAAAG
PmcjASalI	GGTGGTGTCGACTCATACTATTTCCATCAAATAAG
mcjDBamHIR	GGTGGTGGATCCCCGTTTTCCAAGCGACTCCC

Table S2. Sequences of the promoter variants.

Variant	Sequence (5′ to 3′)
PmcjA300	ATTTTGCACACTCCCTCCTGTAGTTATGCCTTTACACTCA<br>ATTGGAATAATAAAACTTCTGAATTTATCTACAAGCTAA<br>ATTTTTTTCTTAATTGATGGCTAAATATTCTGAAATAATTA<br>GAAAAATGTATAAAAATCCAAAATATTGTACTAAATTTG<br>ACCACTTTTGCAGATTGATTAGTTTATGGATGTTTGTATCT<br>AAATGATTTTATTGATAAATTACTAAAGCGTAATGATTAT<br>TGAT**CTCAATTGTATTTTGTGCTAATAAAATTCTAACA**<br>**GAA**GGACGTGAGGTTCCTCTGTAAAAATCATCATACTAT<br>TTCCATCAAAT
PmcjA240	ATTTTGCACACTCCCTCCTGTAGTTATGCCTTTACACTCA<br>ATTGGAATAATAAAACTTCTGAATTTATCTACAAGCTAA<br>ATTTTTTTCTTAATTGATGGCTAAATATTCTGAAATAATTA<br>GAAAAATGTATAAAAATCCAAAATATTGTACTAAATTTG<br>ACCACTTTTGCAGATTGATTAGTTTATGGATGTTTGTATCT<br>AAATGATTTTATTGATAAATTACTAAAGCGTAATGATTAT
PmcjA100	GCGTAATGATTATTGAT**CTCAATTGTATTTTGTGCTAAT**<br>**AAAATTCTAACAGAA**GGACGTGAGGTTCCTCTGTAAAA<br>ATCATCATACTATTTCCATCAAAT

PmcjA89	TTGAT**CTCAATTGTATTTTGTGCTAATAAAATTCTAACA**<br>**GAA**GGACGTGAGGTTCCTCTGTAAAAATCATCATACTAT<br>TTCCATCAAAT
PmcjA87	GCGTAATGATTATTGAT**CTCAATTGTATTTTGTGCTAAT**<br>**AAAATTCTAACAGAA**GGACGTGAGGTTCCTCTGTAAAA<br>ATCATCATAC
PmcjA74	GCGTAATGATTATTGAT**CTCAATTGTATTTTGTGCTAAT**<br>**AAAATTCTAACAGAA**GGACGTGAGGTTCCTCTGTA
PmcjA69	GCGTAATGATTATTGAT**CTCAATTGTATTTTGTGCTAAT**<br>**AAAATTCTAACAGAA**GGACGTGAGGTTCCT
PmcjA63	GCGTAATGATTATTGAT**CTCAATTGTATTTTGTGCTAAT**<br>**AAAATTCTAACAGAA**GGACGTGAG
PmcjA69-35null	**CTCAATTGTATTTTGTGCTAATAAAATTCTAACAGAA**G<br>GACGTGAGGTTCC
PmcjA54	GCGTAATGATTATTGAT**CTCAATTGTATTTTGTGCTAAT**<br>**AAAATTCTAACAGAA**

Note: the minimum shared sequence among all variants, excluding PmcjA240, is shown in bold. The -35 and -10 promoter elements proposed in this study are underlined.

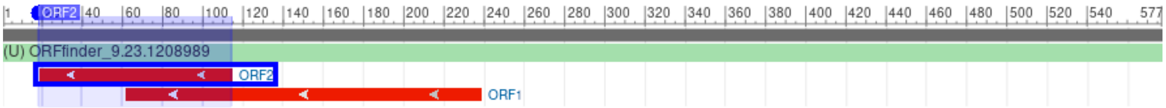

Figure S1. Overlapping ORFs downstream *PmcjA*. The identified open reading frames by the NCBI ORF Finder tool are shown. ORF1: *mcjA*; ORF2: *mcjX*.

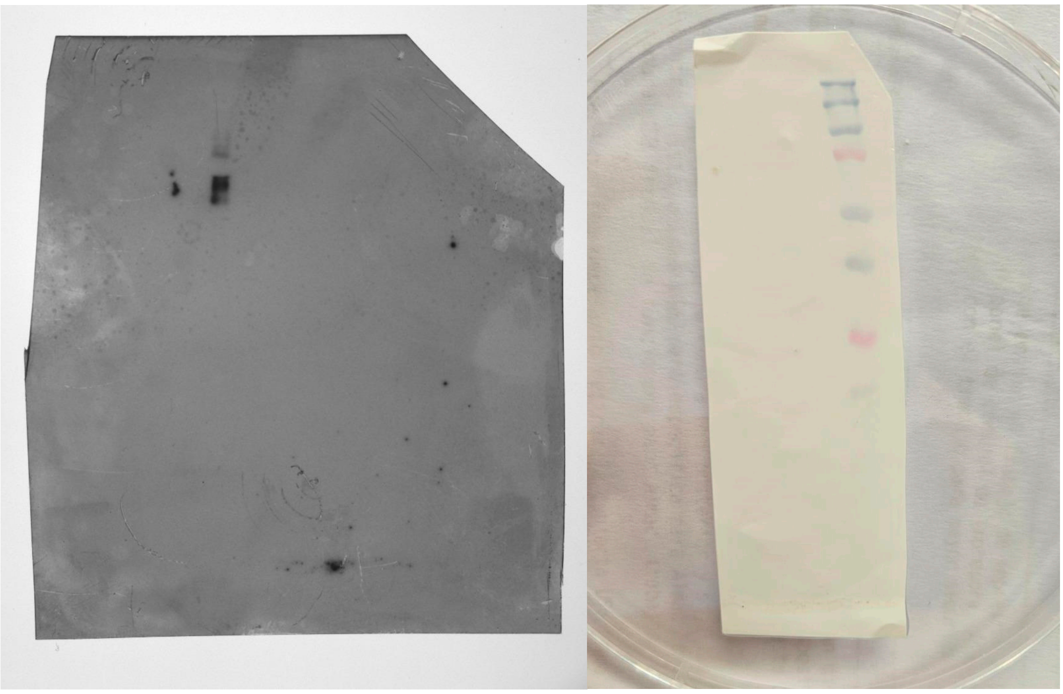

Figure S2. Western-blot of McjX-polyHis is shown in the left panel, whereas molecular weight marker is shown in the right panel.

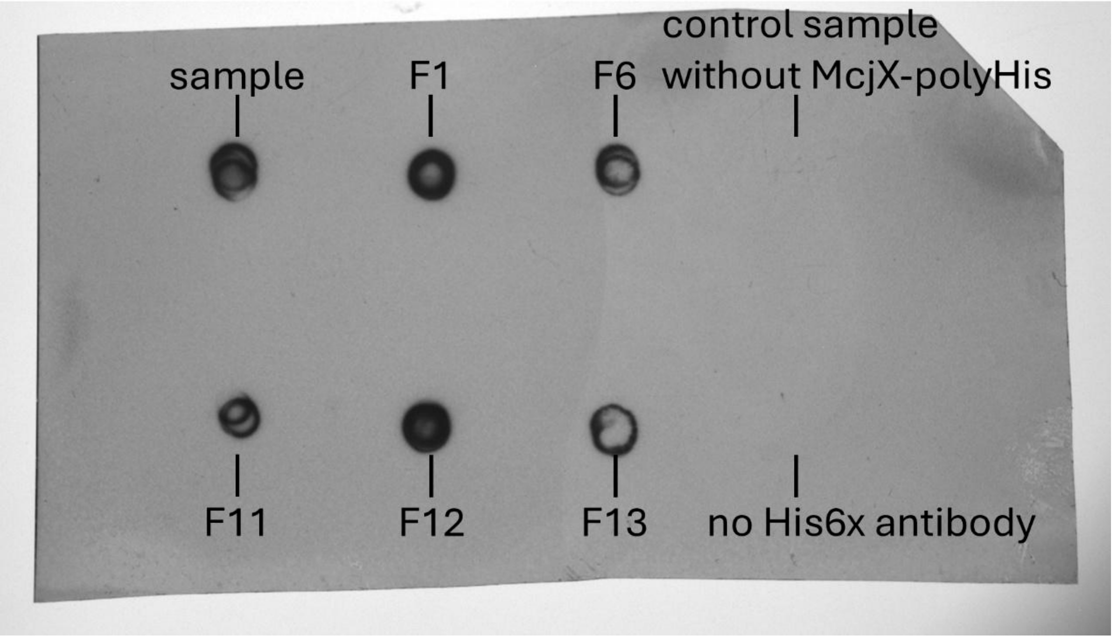

Figure S3. Dot-blot analysis of McjX-polyHis fusion. The bacterial extract from pCHm3H10C-mcjX-bearing *E. coli* DH5α is denoted as "sample". F1 to F6 correspond to the washing steps after loading the Ni-NTA column with the sample. F11-F13 correspond to the elution of McjX-polyHis with 300 mM imidazole.

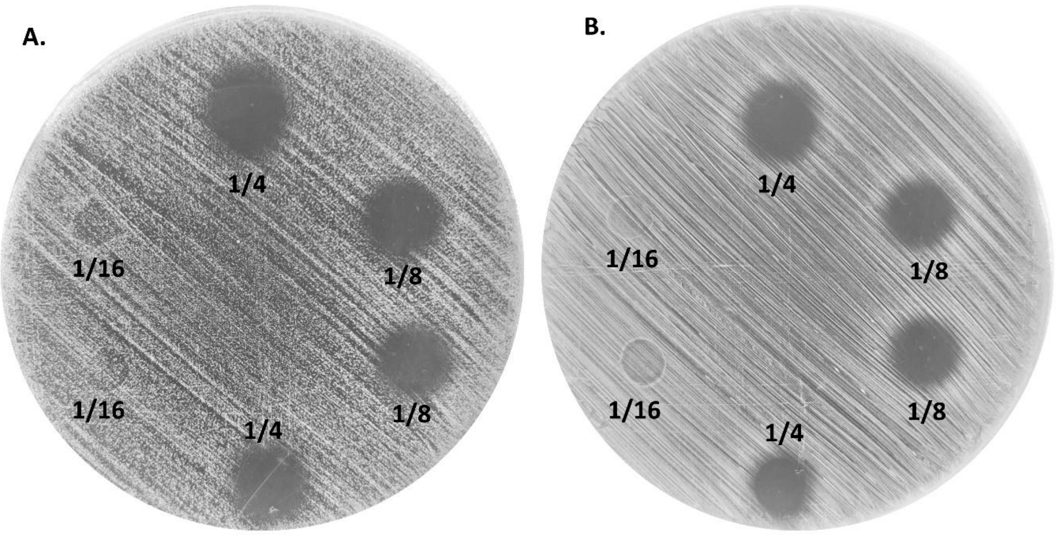

Figure S4. Activity of MccJ25 on *E. coli* BL21 bearing the pACYCDuet-mcjX plasmid plated onto (A) LB with 0.6% glucose and (B) LB containing 50 µM IPTG. Dilutions of MccJ25 were prepared and aliquots were seeded onto LB agar plates previously swabbed with suspensions of 10^8 cells/ml of *E. coli* BL21 pACYCDuet-mcjX. Antimicrobial activity was detected as clear halos of growth inhibition after incubating the plates for 16 h at 37ºC.

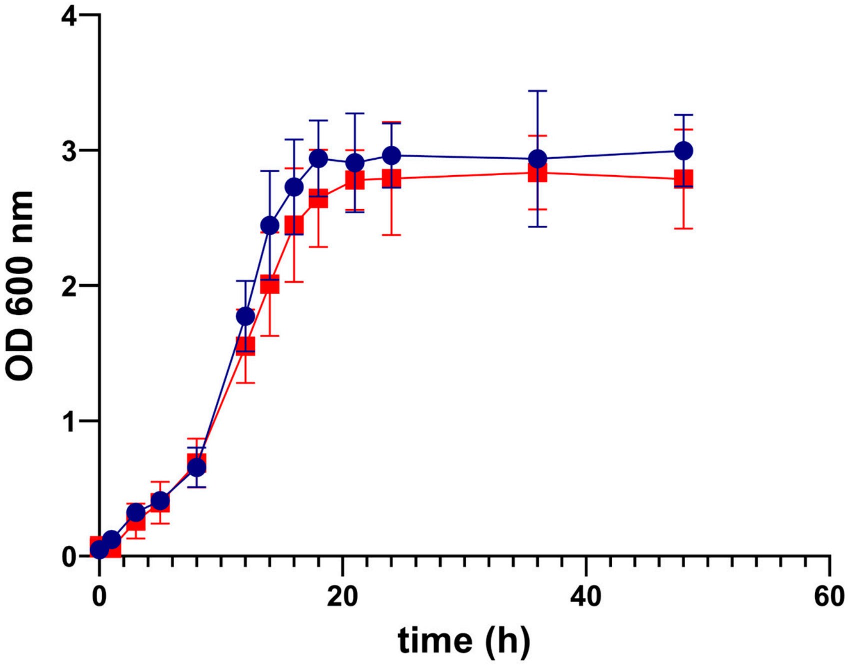

Figure S5. Bacterial growth in LB broth: *E. coli* pUC18 (●) and *E. coli* pTUC346woX (■). Experiments were performed in at least three independent biological replicates. Data are presented as mean ± standard deviation.

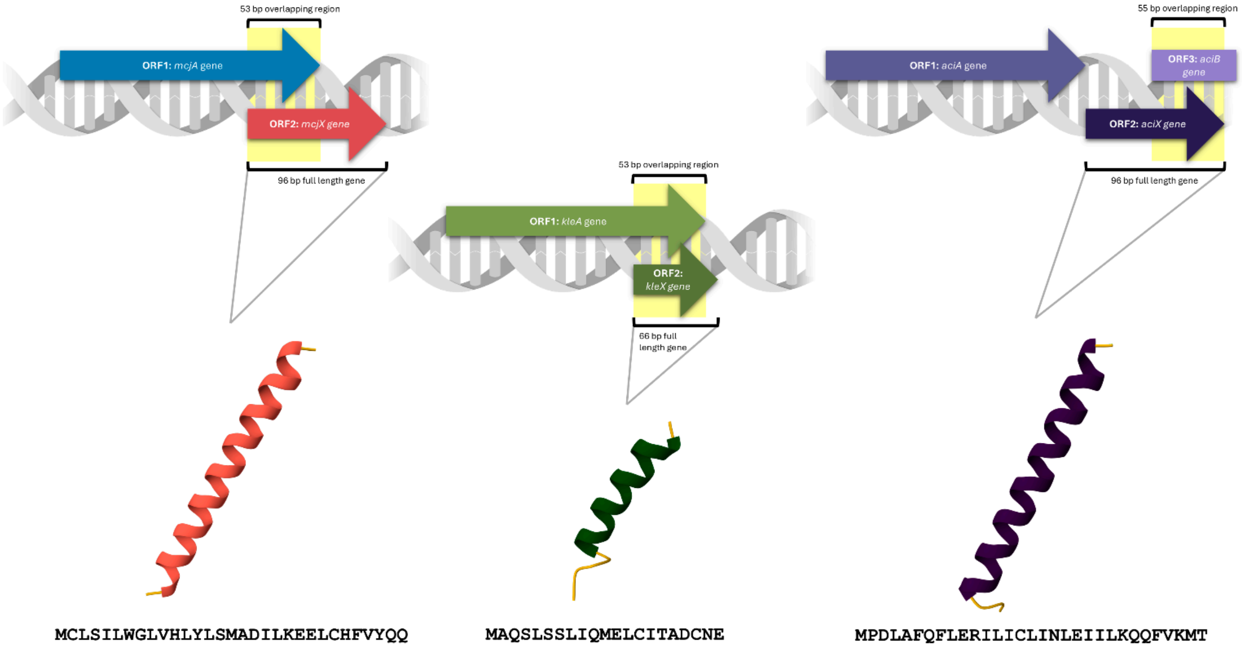

Figure S6. Putative ORFs in klebsidin and acinetodin biosynthetic clusters. Two newly identified ORFs, designated *kleX* and *aciX*, are predicted to encode peptides that form α-helical secondary structures. *kleX* is depicted in green and *aciX* in violet, matching the colors of their predicted structures by AlphaFold. The corresponding amino acid sequence for each peptide is provided at the bottom. All the ORFs are oriented in the same direction to facilitate comparison.
